# Antenatal depression among women with gestational diabetes mellitus: a pilot study

**DOI:** 10.1186/s12978-022-01374-1

**Published:** 2022-03-19

**Authors:** Sa’dia Tasnim, Farzana Mahzabin Auny, Yasseer Hassan, Robana Yesmin, Ismat Ara, Mohammad Sarif Mohiuddin, Mark Mohan Kaggwa, David Gozal, Mohammed A. Mamun

**Affiliations:** 1 Department of Immunology, Bangladesh University of Health Sciences, Darus Salam Road, Dhaka, 1216 Bangladesh; 2 Department of Public Health, Global Communicable Disease, University of South Florida, Tampa, FL 33620 USA; 3 Director General of Health Services, Ministry of Health and Family Welfare, Mohakhali, Dhaka 1212 Bangladesh; 4 Department of Nutrition and Biochemistry, National Institute of Preventive and Social Medicine, Mohakhali, Dhaka 1212 Bangladesh; 5 Diabetes and Obesity Research Center, NYU Langone Hospital—Long Island, Long Island, New York 11501 US; 6 Department of Psychiatry, Faculty of Medicine, Mbarara University of Science and Technology, 1410, Mbarara, Uganda; 7 African Centre for Suicide Prevention and Research, 379, Mbarara, Uganda; 8 Department of Child Health, The Child Health Research Institute, The University of Missouri School of Medicine, Columbia, MO 65201 USA; 9 Department of Public Health, Daffodil International University, Dhaka, 1341 Bangladesh; 10 Department of Public Health and Informatics, Jahangirnagar University, Savar, Dhaka 1342 Bangladesh; 11 CHINTA Research Bangladesh, Gerua, Dhaka, 1342 Bangladesh

**Keywords:** Antenatal depression, Body mass index, Diabetes and depression, Gestational diabetes mellitus, Maternal depression, Pregnancy and depression, Reproductive health

## Abstract

**Background:**

Gestational diabetes mellitus (GDM) is quite prevalent in low- and middle-income countries, and has been proposed to increase the risk of depression. There is only a prior study assessing antenatal depression among the subjects with GDM in the Bangladesh, which leads this study to be investigated.

**Objective:**

To determine the prevalence of depressive symptoms and potential associations among pregnant women diagnosed with GDM.

**Methods:**

A cross-sectional study was carried out among 105 pregnant women diagnosed with GDM over the period of January to December 2017 in 4- hospitals located in two different cities (Dhaka and Barisal). A semi-structured questionnaire was developed consisting of items related to socio-demographics, reproductive health history, diabetes, anthropometrics, and depression.

**Results:**

Mild to severe antenatal depression was present in 36.2% of the subjects (i.e., 14.3%, 19% and 2.9% for mild, moderate and severe depression, respectively). None of the socio-demographic factors were associated with depression, but the history of reproductive health-related issues (i.e., abortion, neonatal death) and uncontrolled glycemic status were associated with the increased risk of depressive disorders.

**Conclusions:**

GDM is associated with a high prevalence of depressive symptoms, which is enhanced by poor diabetes control. Thus, in women presenting with GDM, screening for depression should be pursued and treated as needed.

## Plain English Summary

Pregnancy is a highly stressful period in a woman's life that can also be associated with mental health problems such as depression. Depression is reported in about 16% of pregnant women whereas and such prevalence can double in LMICs (e.g., Bangladesh). In addition, gestational diabetes mellitus (GDM) has emerged as a common condition affecting approximately 10% of all pregnancies. GDM has also been associated with adverse mental health outcomes, particularly depression. GDM women with antenatal depression are not only at increased risk of poorer quality of life, but are also at increased risk of adverse pregnancy and fetal outcomes, particularly in LMIC. This study investigates the prevalence of depressive symptoms among Bangladeshi pregnant women diagnosed with GDM. It is found that depression was detectable in 36.2% subjects. In addition, a history of reproductive health-related issues (i.e., abortion, neonatal death) and uncontrolled glycemic status were associated with increased risk of depressive disorders. Considering the negative effects of both GDM and depression on pregnancy-related outcomes, early screening of these conditions should be pursued, preferably once every trimester over the duration of the pregnancy.

## Introduction

Pregnancy is a highly stressful time in a woman's life and is often associated with anxiety and depression. Fear of fetal deformities, economic concerns, and motherhood expectations are the common sources of anxiety that may ultimately lead to depression [1]. According to the WHO, the prevalence of depression in developing countries is around 15.6% during pregnancy [2]. However, a systematic review of studies conducted in developed and low-income countries reported the prevalence of gestational depression as ranging between 5 to 30% for developed economies [1, 3, 4] and 15.6% to 31.1% for low- and middle-income countries (LMICs) like Bangladesh [5]. These estimates varied according to ethnicity, history of miscarriage, issues related to medically assisted pregnancy, ambivalent attitude about the pregnancy, and socioeconomic condition of the women [1, 3, 5–7]. Depression is an abnormal psychological state that is usually characterized by excessive or long-term decreased mood and loss of interest in enjoyable activities, and reduced quality of life [8, 9], all of which can lead to a vast array of pernicious consequences for both mother and child.

In recent years, gestational diabetes mellitus (GDM) has emerged as a common condition during pregnancy [10]. Indeed, one in 10 pregnancies is estimated to be associated with diabetes, whereas 90% of these cases are reported as GDM [11, 12]. Furthermore, the risk of GDM can rise to 30% of all pregnancies if obesity is present [11], with the majority of cases (87%) being reported from LMIC [12]. The prevalence of GDM has been progressively increasing in Bangladesh compared to other South-East Asian countries [13], with pooled estimates indicating a prevalence of about 8.0% [14–16]. The presence of GDM increases the risk of adverse effects on both the mother and child. The most common complications include an increased risk of fetal loss as well as postpartum development of type 2 diabetes in the mother [11, 17, 18].

GDM has also been associated with adverse mental health outcomes, particularly depression. GDM subjects with antenatal depression are not only at increased risk of poorer quality of life [19], but are also at increased risk of adverse pregnancy and fetal outcomes, particularly in LMICs [20–22]. Considering the potential negative consequences of GDM and gestational depression and the scarcity of information regarding these issues in Bangladesh [23], the present study was undertaken to investigate the prevalence of depressive symptoms and potential associations among Bangladeshi pregnant women diagnosed with GDM.

## Methods

### Study design and participants

A cross-sectional study was conducted to assess the prevalence of depressive symptoms and potential associations among Bangladeshi pregnant women diagnosed with GDM within January to December 2017 in two different cities (Dhaka and Barisal). Two hospitals from each city were included based on the criteria of having adequate facilities to deal with GDM patients and availability of patients seeking medical assistance from remote areas and those who had GDM-related complications. Therefore, it is assumed that the vast majority, if not all pregnant women who were at risk, suspected to suffer from GDM, or those formally diagnosed as GDM patients would come to these hospitals for their treatment and antenatal check-ups. The survey was carried out in the outpatient departments of the four hospitals, namely (i) BIRDEM General Hospital (Dhaka), (ii) BSMMU Hospital (Dhaka), (iii) Sher E Bangla Medical College Hospital (Barisal), and (iv) Advocate Hemayet Uddin Ahmed Diabetic & General Hospital (Barisal).

### Data collection approach

Before the onset of the data acquisition interviews, the semi-structured questionnaire was pilot tested on a total of 10 respondents to ascertain it was easily understandable by all interviewees. After implementing changes based on the feedback from the pre-testing phase, data were collected from the respondents through face-to-face interviews conducted in Bangla, the native language of both the research team and the participants. However, respondents were identified by purposive sampling after compiling selection criteria, which included: (i) pregnant women diagnosed with GDM by the hospital physician and (ii) women who were willing to participate. Participants were excluded from the study if they (i) had pre-gestational diabetes and comorbid conditions, (ii) were severely ill or unable to participate, or (iii) were not willing to participate. A total of 105 interviews were ultimately included for analyses.

### Ethical considerations

A formal ethical approval of the study was obtained from the Institutional Review Board at the National Institute of Preventive and Social Medicine, Dhaka, Bangladesh (ethical clearance approval number: NIPSOM/IRB/2017/245). Participation in this study was absolutely voluntary. Potential subjects were informed that they have the right to refuse to respond to any of the entire set of interview questions and that they also have the right to withdraw from an ongoing interview. Subjects were also clearly informed about the confidentiality of their data and provided complete assurance that all information would be kept confidential and their names or anything which can identify them would not be published or exposed anywhere. Participants had to provide consent by signature or thumb impression.

### Measures

A semi-structured questionnaire was developed in Bangla consisting of questions related to (i) socio-demographics, (ii) reproductive health, (iii) diabetes, (iv) anthropometrics, and (iv) depression. Permission for using the depression assessment instrument was granted by the developer of the Montgomery-Asberg Depression Rating Scale (MADRS). A short description of all variables included in this study is given below.

#### Socio-demographic factors

The basic socio-demographic information of the participants, such as age, residence, religion, family type, family income, family expenditure, occupation, and education, were documented. In addition, the occupation and education of the participants’ spouses were explored.

#### Reproductive Health-related factors

Data on reproductive health-related issues such as the age of marriage, duration of married status, age of first pregnancy, the total number of pregnancies, total number of children, age of the last child born, etc., were collected. In addition, a history of (i) intrauterine death, (ii) abortion, (iii) dilation and curettage, and (iv) neonatal death was obtained. Subsequently, a continuous variable was created, compiling all the history-related variables.

#### Diabetes-related factors

A number of factors associated with GDM were collected in this study. First of all, the history of GDM diagnosis and hypertension in the past pregnancy was assessed. Furthermore, family history of diabetes, personal history of hypertension, and status of smoking and smokeless tobacco use were asked. The aforementioned variables were compiled to create a continuous variable on GDM related issues.

#### Glycemic status

To evaluate GDM glycemic control or the current use of glucose-lowering medications [24], recently diagnosed glycemic status data were collected from the participants’ patient charts. Based on the American Diabetes Association diagnostic criteria [25], GDM participants were classified to ‘no control of diabetes’ based on their blood glucose (BG) levels (i) fasting: ≥ 5.3 mmol/l, or (ii) 2 h after breakfast (ABF): ≥ 8.6 mmol/l.

#### Anthropometric measures

Measurements of the height and weight of the participants were performed. For assessing body mass index (BMI), weight in kilos was divided by the square of height in meters. The research assistants measured height and weight. The participants' weight was measured with a digital scale with an accuracy of 0.1 kg; with participants having on light clothing and without shoes. The digital weighing scale measurement accuracy was checked at various stages using standard weights. The height of the participants was measured using a tape with an accuracy of 0.1 cm that was fixed to the wall with a special tool in the different clinics. The participants took off their shoes and heels; buttocks, shoulders, and back of the head touched the wall, and the Frankfort line was parallel to the ground. Later on, the recommended BMI categories were followed: (i) < 18.5 kg/m^2^ = underweight, (ii) 18.5–24.9 kg/m^2^ = normal or healthy weight, (iii) 25.0–29.9 kg/m^2^ = overweight, and (iv) ≥ 30.0 kg/m^2^ = obese [26].

#### Depression scale

Depression was assessed by the 10-item MADRS [27]. Since its development, the scale has been widely validated and used globally, including in Bangladesh [28, 29] and has also been used in GDM patients [23]. The scale contains symptoms related to (i) apparent sadness, (ii) reported sadness, (iii) inner tension, (iv) reduced sleep, (v) reduced appetite, (vi) concentration difficulties, (vii) lassitude, (viii) inability to feel, (ix) pessimistic thoughts, and (x) suicidal thoughts [27]. Based on the five-point Likert scale (0 to 6), the total score of the scale ranges from 0 to 60 points. Like in previous studies [23, 28, 30], the MADRS scores are categorized into 4 groups, healthy (0–12 points), mild depression (13–19 points), moderate depression (20–34 points) and severe depression (35–60 points) [27].

### Statistical analysis

After data collection, individual questionnaires were edited for completion and consistency. Only fully completed questionnaires were entered into the statistical software (SPSS 22, IBM Corporation, Chicago, IL, USA) for analysis. Descriptive statistics (e.g., percentage, frequencies, mean and standard deviations) were used to describe the data. Inferential statistics (e.g., ANOVA tests, independent t-tests, etc.) were performed to identify significant associations of the studied variables with depression as the outcome variable. A two-tailed *p*-value of < 0.05 and 95% confidence interval was considered statistically significant.

## Results

**Fig. 1:**
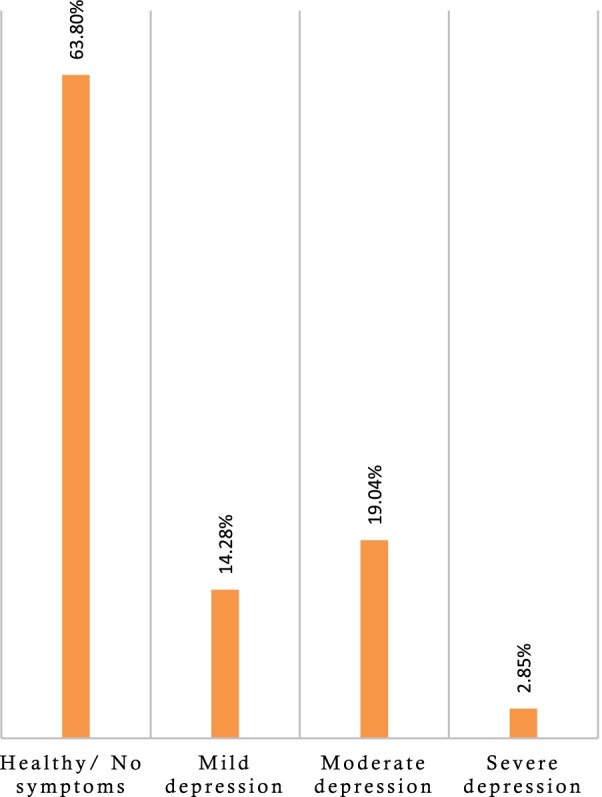
Distribution of the depression of the subjects with GDM

The socio-demographic characteristics of the participants are presented in Table 1, whereas Tables 2 and 3 show reproductive health history and GDM-related variables, respectively. Of the 105 women with GDM, 60.0% were in the 26–33-year-old group, and their mean age was 28.98 (± 4.87) years. Most of them were Muslim (88.6%), urban residents (75.2%), and housewives (67.6%) (Table 1). Only 25 women reported having a personal monthly income (42,000 ± 17,795 BDT). The mean total family income of the participants was 57,028 ± 33,860 BDT, whereas 26,400 ± 13,945 BDT was the average reported monthly expenditure (Table 4). About 36.2% of the subjects with GDM reported suffering from mild to severe levels of depression (Fig. 1). However, bivariate analyses showed no significant associations between socio-demographic factors and depression levels (Table 1).

**Table 1:** Distribution of the Socio-demographic variables with depression

Variables	Total sample				
	n (%)	Mean	SD	f/t-value	*p*-value
Age group					
18–25 years	23; 21.9%	12.78	8.61	0.265	0.768
26–33 years	63; 60.0%	12.83	9.51		
34–41 years	19; 18.1%	11.16	7.40		
Religion					
Muslim	93; 88.6%	12.06	8.91	2.091	0.151
Hindu	12; 11.4%	16.00	8.57		
Residence					
Urban	79; 75.2%	12.63	9.13	0.056	0.814
Rural	26; 24.8%	12.15	8.41		
Family type					
Joint	43; 41.0%	12.93	8.39	0.157	0.693
Nuclear	62; 59.0%	12.22	9.32		
Education					
Up to primary	21; 20.0%	14.00	10.18	0.365	0.695
Secondary	40; 38.1%	12.25	7.57		
Higher	44; 41.9%	12.04	9.53		
Occupation					
Housewife	71; 67.6%	12.45	8.47	0.480	0.620
Service holder	14; 13.3%	10.86	9.47		
Others	20; 19.0%	13.90	10.29		
Husband’s education					
Up to primary	7; 6.7%	11.71	7.95	0.938	0.395
Secondary	30; 28.6%	14.40	10.09		
Higher	68; 64.8%	11.76	8.45		
Husband’s occupation					
Service holder	43; 41.0%	12.65	9.41	2.441	0.069
Business man	31; 29.5%	15.29	8.76		
Stay in abroad	12; 11.4%	8.00	5.12		
Day labor	19; 18.1%	10.53	8.84

**Table 2:** Distribution of the reproductive health-related variables and association with depression

Variables	Total sample				
	n (%)	Mean	SD	f/t-value	*p*-value
History of intrauterine death					
No	100; 95.2%	12.38	8.95	0.474	0.493
Yes	5; 4.8%	15.20	8.79		
History of abortion					
No	74; 70.5%	11.27	8.49	5.067	0.027
Yes	31; 29.5%	15.48	9.34		
History of dilation and curettage					
No	89; 84.8%	12.02	8.57	1.790	0.184
Yes	16; 15.2%	15.25	10.55		
History of neonatal death					
No	93; 88.6%	11.76	8.33	6.048	0.016
Yes	12; 11.4%	18.33	11.37

**Table 3:** Distribution of the diabetes-related variables and association with depression

Variables	Total sample				
	n (%)	Mean	SD	F/t-value	*p*-value
History of GDM in past pregnancy					
No	76; 72.4%	12.68	9.14	0.099	0.754
Yes	29; 27.6%	12.07	8.44		
Family history diabetes mellitus					
No	39; 37.1%	11.95	8.48	0.248	0.620
Yes	66; 62.9%	12.85	9.22		
History of hypertension					
No	91; 86.7%	12.59	8.95	0.053	0.818
Yes	14; 13.3%	12.00	9.05		
History of hypertension in past pregnancy					
No	100; 95.2%	12.58	8.92	0.113	0.737
Yes	5; 4.8%	11.20	9.86		
Family history of hypertension					
No	58; 55.2%	12.03	8.41	0.373	0.543
Yes	47; 44.8%	13.11	9.57		
Glycemic status					
Both fasting and 2 h ABF BG level are in control	16; 15.2%	9.38	5.30	22.403	< 0.001
Either fasting or 2 h ABF BG level is in control	48; 45.7%	8.33	5.04		
Both fasting and 2 h ABF BG level are not in control	41; 39.0%	18.49	9.98		
Body mass index (BMI)					
Normal	23; 21.9%	12.00	7.38	0.100	0.905
Overweight	44; 41.9%	12.95	9.03		
Obesity	38; 36.2%	12.32	9.79

**Table 4:** Correlations of the continuous variables with depression

Variables	Mean ± SD	Correlation coefficient (*r*)	2-tailed Sig. (*p*)
Age	28.98 ± 4.87	− 0.085	0.390
Monthly personal income	42,000.00 ± 17,795.13	0.074	0.726
Monthly family income	57,028.57 ± 33,860.14	0.017	0.862
Monthly family expenditure	26,400.00 ± 13,945.53	0.033	0.740
Age at marriage	21.49 ± 4.339	− 0.019	0.844
Marriage duration	7.58 ± 5.31	-0.067	0.497
Age of first conceive	23.54 ± 5.31	0.087	0.375
Total number of conceive	2.40 ± 1.31	− 0.203	0.138
Total number of children	1.64 ± 0.85	0.039	0.775
Age of last children	5.96 ± 3.53	0.001	0.994
Total reproductive health related history	0.61 ± 0.75	0.314	< 0.001
Total diabetes related history	1.53 ± 1.07	0.023	0.814
Fasting blood glucose level	5.91 ± 1.34	0.088	0.373
2 h after breakfast glucose (AFB) level	8.52 ± 2.29	0.271	0.005
Both fasting and 2 h AFB glucose level	14.43 ± 3.08	0.239	0.014
Body mass index	28.95 ± 4.91	− 0.061	0.533

Among the participants, 4.8% had a history of intrauterine death, whereas 29.5%, 15.2%, and 11.4% reported a history of abortion, dilation and curettage, and neonatal death, respectively. Subjects reporting a history of abortion and neonatal death were significantly more likely to suffer from depression (f = 5.07, *p* = 0.027 and f = 6.05, *p* = 0.016, respectively) (Table 2). In addition, 0.61 (± 0.75) was the mean score of total reproductive health-related history, which was also significantly correlated with depression (r = 0.314, *p* < 0.001) (Tables 2, 4).

About 27.6% and 4.8% of the subjects were diagnosed with GDM and hypertension in their prior pregnancy, respectively, and 13.3% reported suffering from hypertension during the non-pregnant period. Similarly, 62.9% and 44.8% reported that someone in their family had suffered from diabetes mellitus and hypertension, respectively. Among the participants, 39.0% GDM was not controlled based on both fasting and 2 h after breakfast glycemic levels, and 45.7% were deemed as controlled. In addition, 41.9% were overweight, and 36.2% were obese. However, neither previous pregnancy diabetes nor hypertension history, nor BMI status were significantly associated with depression, but current GDM glycemic status was. In fact, the lesser the GDM control, the more likely subjects were to report depression (f = 22.40, *p* < 0.001) (Tables 3, 4).

## Discussion

The prevalence of severe levels of depressive symptoms among women with GDM was 36.2% and was associated with a history of abortion, neonatal death, and poor GDM glycemic control. This study shows that the presence of GDM, particularly when glycemia is not well-controlled among expectant Bangladeshi mothers, is associated with an increased risk of depression. It is now well established that the presence of antenatal and postpartum depression imposes substantial adverse effects on both mothers and their offspring [17, 31, 32]. In addition, many of the mothers with antenatal depression will also suffer from depression after labor and delivery, with 39% of postnatal depression rates being attributable as being initiated and established during the pregnancy period [31]. Thus, early identification and treatment of antenatally depressed subjects with GDM are critical [33, 34].

Before entertaining the potential implications of the present study, several methodological issues deserve comment. First of all, this was a cross-sectional study which may hinder the ability to infer causal associations. Second, participants were identified from four hospitals and included a relatively small sample size; therefore, generalizability may be limited. Third, this study lacked a control group of participants without GDM, a comparative control group. However, the present study provides important and scarcely available information in the Bangladeshi context, and the findings further reinforce the need to expand the study and identify viable pragmatic interventions to prevent the deleterious consequences of GDM and depression on both mother and child.

The prevalence of all severities of depression was 36.2%, whereas the only prior study assessing depression in Bangladesh reported a prevalence of 18.32% among pregnant women with and without GDM [23]. Furthermore, the investigators reported that a prevalence of 25.92% (12.70%, 5.48% and 0.14% mild, moderate and severe depression, respectively) for antenatal depression was present among women with GDM [23]. Of note, a review article estimated the prevalence of mental disorders in Bangladesh within 6.5 to 31.0% of all adult subjects [35], while the median prevalence of antenatal depression has been estimated to be around 14.7–24.3% globally [17, 31, 36]. Furthermore, the prevalence rates of antenatal depression were 7.4%, 12.8%, and 12.0% in the first, second, and third trimesters, respectively [37]. However, antenatal depression rates as high as 57% have been reported in LMIC such as Bangladesh [3], with a pooled prevalence estimated around 34.0% (compared to 22.7% in middle-income countries) [5]. Depression-related studies considering special situations of pregnant women (for example, gestational diabetes) are somewhat limited in the literature [17, 36]; only a prior study was conducted in Bangladesh [23].

Although many factors related to socio-demographic (e.g., low socioeconomic status, low social support, lower education levels, poor marital relationships etc.), psychological (e.g., psychiatric illness history, stressful life events, exposure to violence etc.), and health (e.g., negative attitude towards pregnancy, negative obstetric history etc.) have been associated with antenatal depression risk [1, 3, 5], the potential contribution of GDM to this risk has only been sporadically examined. However, as suggested by the present study, pregnant women with GDM are at high risk of depression, and such risk is further exacerbated by poor control of their glycemic state.

GDM subjects with a history of reproductive health-related complexities were more likely to be depressed. Such associations have been previously identified, and the present study concurs with such findings [6, 7, 38]. It can be postulated that poor diabetes self-care increases the risk of depression, likely related to the complex nature of diabetes management in LMIC, and the impositions of GDM on lifestyle, particularly when the access to care is sporadic and difficult [33, 39]. Indeed, it is possible that GDM women who have ready access to interventions and medical care for their diabetes (i.e., dietary advice, glucose monitoring, insulin therapy etc.) may be less likely to develop adverse mental health outcomes [40]. Consequently, the inability to establish GDM glycemic control may simply reflect the lack of access to overall care, which may exacerbate the propensity for antenatal depression in these cases.

## Conclusions

In a group of GDM women, 36.2% suffered from depressive symptoms, and the depression severity was enhanced by the presence of underlying poor glycemic control. Considering the known negative impact of GDM and depression on pregnancy-related outcomes, early screening of these conditions should be pursued, preferentially once every trimester over the duration of the gestational period.

## Data Availability

The dataset used and/or analyzed during the current study are available from the corresponding author on reasonable request.

## Abbreviations

ABF: 2H after breakfast
BG: Blood glucose
BMI: Body mass index
GDM: Gestational diabetes mellitus
LMICs: Low-and middle-income countries
MADRS: Montgomery-Asberg Depression Rating Scale
